# Angio-Neurotic Œdema

**Published:** 1907-09-14

**Authors:** James Burnet

**Affiliations:** Lecturer on Practical Materia Medica and Pharmacy; Registrar, Royal Hospital for Sick Children, Edinburgh


### Hospital Clinics.

### ANGIO-NEUROTIC ŒDEMA.

By JAMES BURNET, M.A., M.D., M.R.C.P. Edin., Lecturer on Practical Materia Medica and Pharmacy; Registrar, Royal Hospital for Sick Children, Edinburgh.

The condition under consideration is one of those comparatively rare diseases with which the physician has to deal. It is because of its rarity and the scanty literature on the subject that we know so little regarding its etiology and pathology. Angio-neurotic œdema is to be defined as a circumscribed, non-inflammatory swelling associated with definite sensory changes in the affected part, and occasioned in all probability by a vasomotor neurosis. The site of the œdema varies in different cases, and may be almost any part of the body. The commonest situations, however, are the face, especially the eyelids, cheeks, and lips, the extremities, the throat, and the external genital organs, more particularly the scrotum and vulva. It is conceivable, also, that this form of œdema may be present in connection with the various internal mucous membranes, such as those of the intestines and stomach. The surface of the swollen area is usually red, but is not always tense. It sometimes happens that the œdema varies its position. Thus it may start in the hand, disappear, and then re-appear in some other part, such as the throat. In very rare instances angio-neurotic œdema may be met with involving almost the entire body.

Although at the commencement the area affected may be more or less red in appearance, this redness often disappears, giving place to discoloration of the parts such as is met with after a bruise. In such cases we are sometimes reminded of purpura, as the appearance is not at all unlike. There is very often some disturbance of sensation in the parts affected. Thus there is usually a sense of fullness, and occasionally a throbbing sensation is experienced by the patient. Burning or scalding is also very often complained of. In one of my cases the patient constantly spoke of a curious pricking feeling, which at times disappeared, and was succeeded by a sensation of numbness in the œdematous part. So far as my experience goes there is seldom any actual pain present, although itching is, I think, not altogether unknown in connection with angio-neurotic œdema.

As regards the etiology of this affection very little is known. I have divided the cases as met with clinically into three groups, but this, it must be understood, is a somewhat arbitrary classification, and one which is made more for the sake of convenience in description than because of its scientific accuracy or completeness. The three groups are to be described as (1) traumatic, (2) neurotic, and (3) climacteric. I am convinced that practically every case of angio-neurotic œdema can be referred to one or other of these classes. By far the commonest are the neurotic cases, and this affection is certainly much more frequently met with in the female than in the male. It must not be imagined, however, that males are altogether exempt, for I have met with two cases where the subjects were boys of school age. The traumatic form is usually based upon a neurotic heredity and constitution, while, of course, in the climacteric variety there is always an element of nervous instability. I think angio-neurotic œdema is practically never met with in those who are not of a nervous temperament. It is certainly favoured by the presence of anæmia, and by mental worry or overwork. So far as I can discover it is not a hereditary disease, although I should not like to dogmatise on this point.

The pathology of angio-neurotic œdema is somewhat obscure, owing to the lack of clinical material at the disposal of observers. We do know, however, that it is the cellular tissue of the skin and mucous membranes which are principally concerned. Its relation to urticaria is very interesting. I have found on more than one occasion that the œdema was really associated with a genuine urticarial eruption, while one case presented a severe erythema. Some observers, indeed, regard angio-neurotic œdema as being only an exaggerated form of giant urticaria. Against this view, however, is to my mind the almost insuperable objection that giant urticaria is not necessarily of neurotic origin. Angio-neurotic œdema is in all probability a vaso-motor and tropho-neurosis, but how it is actually induced cannot be so readily explained. On the whole I am inclined to believe that in many cases, if not in all, it is an exaggerated form of erythema, and may indeed have some relation to that curious condition known as erythema nodosum. In fact the whole question as to the exact pathology of angio-neurotic œdema is, and must be, at the present time purely problematic. Nothing is definitely known, and only continued clinical observation will serve to bring about the acquisition of definite knowledge regarding this somewhat anomalous disease.

In a few cases the condition presents certain complications. Of these the commonest by far are gastro-intestinal disturbances, such as nausea, vomiting, and even colic. The only satisfactory explanation which can be given of such complications is that there is present at the same time œdema of the gastro-intestinal mucous membranes. Urinary affections have also been described by various writers. In this connection albuminuria and hæmoglobinuria are worthy of note. It is quite conceivable that the kidney may become involved in this disease, or the mucous membrane of the bladder may very readily be œdematous and so give rise to hæmorrhage. Angina pectoris as a complication must indeed be extremely rare, still it has been mentioned by those acquainted with this disease. The presence of urticaria or of erythema in the patient who is suffering from angio-neurotic œdema must, I think, be regarded as of special significance. In fact, the relation of urticaria to angio-neurotic œdema is a somewhat close one. If we consider for a moment the pathology of urticaria we must be struck by this. The lesion of urticaria is due to a vasomotor neurosis which in turn leads to œdema of the skin area involved, together with a certain amount of serous exudation; and in angio-neurotic œdema the pathological changes are very similar. Whether or not, however, it is but a form of urticaria I am not prepared to state. There are to me insuperable difficulties in believing that it is. On the other hand, the form of erythema known as erythema fugax suggests to my mind a resemblance to angio-neurotic œdema, although some observers regard this form of erythema as merely a special modification of urticaria.

The clinical classification of the varieties of angio-neurotic œdema which I have suggested forms a fairly satisfactory basis on which to give a few examples of this disease as I have met with it from time to time. In the first place the traumatic form is not, in my opinion, so common as the other two—namely, the purely neurotic and the climacteric. I have, as a matter of fact, only met with two examples of the traumatic variety. One of these I have already described in the "International Clinics" (vol. iv., thirteenth series). As the case is a very typical example, I may once more refer to it in this place. The patient was a girl of eighteen years. She came complaining that her left foot was so badly swollen that she could hardly walk. She said a needle had lodged in the sole of the foot six years previously, and had never been extracted. It did not, however, cause any great pain, and the patient was able to walk about perfectly well after a few weeks' rest. From that time she had made repeated attempts to find the needle by pressing her foot between her hands, but she had failed to locate it. When I examined the foot I found the dorsum considerably swollen. Pressure seemed to cause the patient considerable pain, and pitting was readily produced. The swelling was quite circumscribed, and had a distinctly ovoid outline. The patient was anæmic. The teeth were in a very carious state, but beyond this there were no evidences of disease. There was no history of angio-neurotic œdema in the family. In fact the family history was excellent. The patient herself had never been seen by a doctor since babyhood. She was told that the condition was not a serious one, and that it had nothing whatever to do with the needle incident, of which the patient was apparently very much afraid. She was given a mixture containing a small amount of sodium bromide, and was told to apply iced compresses to the swelling. At the end of a week the swelling was found to be greatly diminished, and the burning sensation was no longer present. Ten days later the foot was found to be absolutely normal, and the patient had no subjective symptoms of any kind.

In this particular case I am inclined to believe that the prolonged mental effect of the fixed idea that there was a needle in the foot produced a vasomotor disturbance. At anyrate, as soon as the patient's mind was set at rest the condition was very speedily relieved. In my opinion, the bromide mixture and the iced compresses had little, if anything, to do with the cure.

Of purely neurotic cases I have witnessed several examples. One of the best, however, was that of a young lady who had been married for about eighteen months. She was always very anæmic and nervous. After a sharp attack of appendicitis from which she made a very slow recovery, she had been in the country for nearly two months and appeared to be getting on all right. But on the very day after her return home she suddenly developed a swelling over the right elbow-joint, which became quite stiff. The swelling was situated towards the external aspect of the joint and extended for a short distance upwards. The skin over the swelling was somewhat discoloured. The forearm was swollen along the radial border, and there was also some œdema of the hand, wrist, and fingers of the same side. Over the affected parts of the hand and wrist there were several vesicles of minute size, which at times were very itchy. There was no evidence of rheumatism and no history of traumatism. As a local application I ordered a lotion of ichthyol. This, however, seemed to do little good, so I substituted boracic poultices. In a few days the swelling completely disappeared. Just about this time, however, the patient complained one night of severe pain in the left ear. There was also a very sharp headache. On examination I found intense pain produced by pressure over the left supraorbital notch. Next day, however, the pain in the ear was completely gone and that in the head much easier, but a curious state of matters resulted. The left eye was completely closed, and there was found to be a bluish-red swelling of the upper lid. The conjunctiva was somewhat swollen also. Iced compresses were applied with a very satisfactory result. The swelling did not altogether disappear, however, for about a week. The elbow-joint, though no longer swollen, was still observed to be somewhat discoloured, but there was no pain or stiffness present. This case is one of great interest, as it illustrates the possibility of angio-neurotic œdema being migratory in character.

With reference to the migratory nature of this affection it is well to remember that this very fact may render it extremely dangerous and even fatal. Thus a patient may suffer from angio-neurotic œdema of the hands and recover completely, only to be seized with œdema glottidis, from which he may die suddenly. I well remember a case of this kind. The patient was a servant girl of a highly neurotic temperament. She was subject to "swellings" of the hands, and when I saw her she was suffering from acute œdema of the glottis which had come on quite suddenly one night after the patient had returned home. Fortunately she recovered very quickly under treatment. Another somewhat similar case, however, developed such alarming symptoms that tracheotomy was performed. My opinion is that, although such cases are comparatively rare, they ought to be kept carefully in mind as any patient who is in the habit of having attacks of angio-neurotic œdema may at any moment be threatened with œdema of the pharyngeal or laryngeal mucous membranes and the parts in the neighbourhood. Only prompt treatment can avail in such cases, and neglect for a few hours may seriously endanger the life of the patient.

Angio-neurotic œdema may even be met with in children. The latter, however, are practically always either neurotic themselves or born of neurotic or neurasthenic parents. The best example I have ever seen of this type was that of a boy who periodically presented œdema of the scrotum, alternately with œdema of the face and legs. This condition recurred periodically until the age of seventeen, when he ceased to be attacked. The family history of this patient is very interesting as it shows throughout a strongly marked neurotic tendency. The father was a man who was constantly worrying about trifles. The mother was extremely nervous, and when a young woman was subject to hysterical seizures. One of the patient's brothers suffered from periodical attacks of typical migraine, while another suffered very frequently from nervous dyspepsia. The patient's only sister was of a distinctly hysterical temperament.

The climacteric variety of angio-neurotic œdema has a special interest, as it forms one of a series of vasomotor disturbances common to patients at this time of life.

The face, in my experience, is the part of the body most frequently involved. The cheeks are flushed and swollen, and at times the swelling may involve the lips. Curiously enough such swelling of the lips and face may be observed in women who are pregnant and who yet have no albumen present in the urine. The case I referred to under the neurotic type of œdema in which the elbow joint was first involved and then the eyelids, suffered in this way during her first pregnancy. The hands and face, especially the cheeks, eyes, and lips, were very much swollen during the last two months, and this swelling did not disappear for nearly a fortnight after the child was born. In the angio-neurotic œdema, which occurs during the climacteric, there is often very considerable burning heat and itching present. The patient may be in great distress, and be unable to sleep on account of the intense itching. In such cases I think the external application of ichthyol lotions, or a lotion containing bismuth, zinc, and boracic acid, is often very soothing. A smart purge is also to be recommended, as there is usually a considerable degree of constipation present in such cases.

As regards the diagnosis of angio-neurotic œdema, the absence of subjective symptoms is usually considered of very great significance, but this has not been my experience. Itching is certainly often present, while I have remarked on several occasions that the patient complained of a burning sensation in the affected part. On the whole I think the association of a circumscribed swelling with a neurotic history and the absence of any definite cause is significant. If the swelling comes on suddenly in the midst of perfect health, then the diagnosis is fairly certain. It must be remembered that angio-neurotic œdema may be migratory in character, as I have already pointed out, and that it is much more common in the female than in the male. Children, however, may be attacked, and this fact must never be forgotten.

The prognosis is good, but the risk of the œdema spreading to the throat is a grave one, and death may thus be brought about if relief is not speedily obtained.

So far as treatment is concerned, I think more depends on the ability of the physician to reassure the patient than on the actual remedies used. As local applications I think sedatives are best, and iced compresses, boracic acid, or bismuth lotions are usually sufficient. Occasionally ichthyol is of service, but I have known it fail. Internally I prefer small doses of sodium bromide, although it is doubtful if it acts in any other way than as a placebo. Cold douching of the spine is often of service in hysterical cases, and general tonics are always indicated. Constipation and anæmia must be combated, and daily outdoor exercise prescribed. The diet ought to be carefully regulated, and too much red meat and stimulants avoided. Otherwise I do not think more need be attempted. These cases usually recover in a very short time, provided the physician in attendance knows how to deal with patients of a neurotic temperament.

